# Genotype-phenotype relations of the von Hippel-Lindau tumor suppressor inferred from a large-scale analysis of disease mutations and interactors

**DOI:** 10.1371/journal.pcbi.1006478

**Published:** 2019-04-03

**Authors:** Giovanni Minervini, Federica Quaglia, Francesco Tabaro, Silvio C. E. Tosatto

**Affiliations:** 1 Department of Biomedical Sciences, University of Padova, Padova, Italy; 2 CNR Institute of Neuroscience, Padova, Padova, Italy; Icahn School of Medicine at Mount Sinai, UNITED STATES

## Abstract

Familiar cancers represent a privileged point of view for studying the complex cellular events inducing tumor transformation. Von Hippel-Lindau syndrome, a familiar predisposition to develop cancer is a clear example. Here, we present our efforts to decipher the role of von Hippel-Lindau tumor suppressor protein (pVHL) in cancer insurgence. We collected high quality information about both pVHL mutations and interactors to investigate the association between patient phenotypes, mutated protein surface and impaired interactions. Our data suggest that different phenotypes correlate with localized perturbations of the pVHL structure, with specific cell functions associated to different protein surfaces. We propose five different pVHL interfaces to be selectively involved in modulating proteins regulating gene expression, protein homeostasis as well as to address extracellular matrix (ECM) and ciliogenesis associated functions. These data were used to drive molecular docking of pVHL with its interactors and guide Petri net simulations of the most promising alterations. We predict that disruption of pVHL association with certain interactors can trigger tumor transformation, inducing metabolism imbalance and ECM remodeling. Collectively taken, our findings provide novel insights into VHL-associated tumorigenesis. This highly integrated *in silico* approach may help elucidate novel treatment paradigms for VHL disease.

## Author summary

Cancer is generally caused by a series of mutations accumulating over time in a healthy tissue, which becomes re-programmed to proliferate at the expense of the hosting organism. This process is difficult to follow and understand as events in a multitude of different genes can lead to similar outcomes without apparent cause. The von Hippel-Lindau (VHL) tumor suppressor is one of the few genes harboring a familiar cancer syndrome, i.e. VHL mutations are known to cause a predictable series of events leading cancer in the kidneys and a few selected other tissues. This article describes a large-scale analysis to relate known VHL mutations to specific cancer pathways by looking at the molecular interactions. Different cancer types appear to be caused by mutations changing the surface of specific parts of the VHL protein. By looking at the VHL interactors involved, it is therefore possible to identify other candidate genes for mutations leading to very similar cancer types.

## Introduction

Familial cancers are rare, accounting for about 5–10% of all cancers [1–3] and generally characterized by inherited inactivation of important tumor suppressors. Inherited tumors represent a valuable source of information about the mechanisms driving cancerogenesis. These cancers are associated to mutations of known genes, allowing the formulation of clear genotype-phenotype correlations in many cases. The von Hippel-Lindau (VHL) syndrome is a familial disorder characterized by a predisposition to develop several different benign and malignant tumors, such as retinal- and cerebellar-hemangioblastoma, pheochromocytoma, paraganglioma, nonfunctioning pancreatic neuroendocrine tumors (pNETs) and renal cell carcinoma (RCC) [4–8]. VHL syndrome arises from pathogenic inactivation of the von Hippel-Lindau tumor suppressor gene located on chromosome three [4,9], which codes for the homonymous pVHL protein. pVHL is mainly known to act as substrate recognition component of a protein complex [10] formed together with elongin-B, elongin-C and cullin-2 (VCB) [10,11], possessing ubiquitin ligase E3 activity towards the HIF-1α transcription factor [12,13]. Association between pVHL and HIF-1α is oxygen-dependent and triggered through hydroxylation by the PHD (prolyl hydroxylase domain containing) enzyme family of two HIF-1α proline residues [10,12,14,15]. PHD activity is itself inhibited under hypoxia, allowing HIF-1α to escape degradation and translocate to the nucleus where it activates hypoxia-dependent target genes. Clinically, VHL disease is classified as Type 1 or Type 2 depending on clinical manifestations in patients [16]. Type 1 includes patients harboring either truncating mutations or deletions yielding a dysfunctional pVHL presenting a wide spectrum of different cancers but lacking pheochromocytoma. Type 2 is more genetically divergent, characterized by missense mutations and includes patients developing pheochromocytoma [16]. Type 2A presents pheochromocytoma and other typical VHL manifestations (e.g. cysts) except RCC (renal cell carcinoma). Type 2B covers almost the entire spectrum of VHL manifestations including aggressive RCC, while type 2C only develops isolated pheochromocytoma. Although routinely used for the initial assessment of VHL patients, this classification can generate ambiguous assignments. Clinical VHL manifestation is frequently variable, with different phenotypes in different families or even in the same family [17]. Several different functions were attributed to pVHL in addition to its role in HIF-1α degradation in light of these variable phenotypes. pVHL has been reported to associate and promote p53 stabilization upon MDM2 inhibition [18], mediate assembly and regulation of the extracellular matrix (ECM) [19–22], regulate cell senescence [23] and apoptosis [24,25] as well as playing a role in regulating oxidative stress gene transcription response [26]. pVHL is also known to harbor at least three different binding surfaces [27] which are thought to be involved in multiple protein-protein interactions. While hundreds of different pVHL protein-protein associations are described [27–29], whether and how these HIF-independent pVHL contribute to VHL tumorigenesis is largely unknown. Here, we present our efforts in deciphering pVHL function. A thorough investigation of pVHL interactors and binding surfaces was coupled with manual curation of pathogenic mutations. Mutations predicted to impair specific pVHL functions were associated with the corresponding VHL phenotype, while a list of affected pathways was constructed for each phenotype. Our analysis shows that the different phenotypes described in VHL patients correlate with specific structural pVHL perturbations, showing how pVHL interfaces to correlate differentially with specific phenotypes. Our data also show that some HIF1-independent functions of pVHL can be attributed to specific pVHL regions.

## Results

Although the best known pVHL function is its role in degrading the HIF-1α transcription factor [4], a number of HIF-independent functions have also been reported [30,28]. We previously proposed three distinct pVHL interaction interfaces (A, B, and C) [27], engaging in exclusive interactions with different interactors and partially explaining the pVHL binding plasticity. Based on the available pVHL 3D structures, interface A is important for VCB complex formation [31]. Interface B forms the HIF-1α binding-site [10], while interface C participates in the cullin2 interaction [31]. A further interface is defined by an accessory N-terminal tail (surface D) present only in the pVHL30 isoform [29]. As the recently developed VHLdb database [29] collects pVHL interactors and mutations, here we present an analysis of this data to shed light on pVHL functions. VHLdb missense mutations were mapped on the pVHL structure to highlight specific interaction surfaces. Deletions of vast pVHL regions were excluded as they yield a clearly dysfunctional protein. A detailed investigation of pVHL interactors was carried out to assess their ability to explain different VHL phenotypes. Collectively, our analysis covers 59 pVHL interactors and 360 different mutations from 2,195 VHL patients described in 1,670 case reports from 156 scientific papers. These data were used to list the most affected pVHL interactors in VHL disease and their effects were simulated using a Petri net [32].

### pVHL mutation hot spots

**Fig 1:**
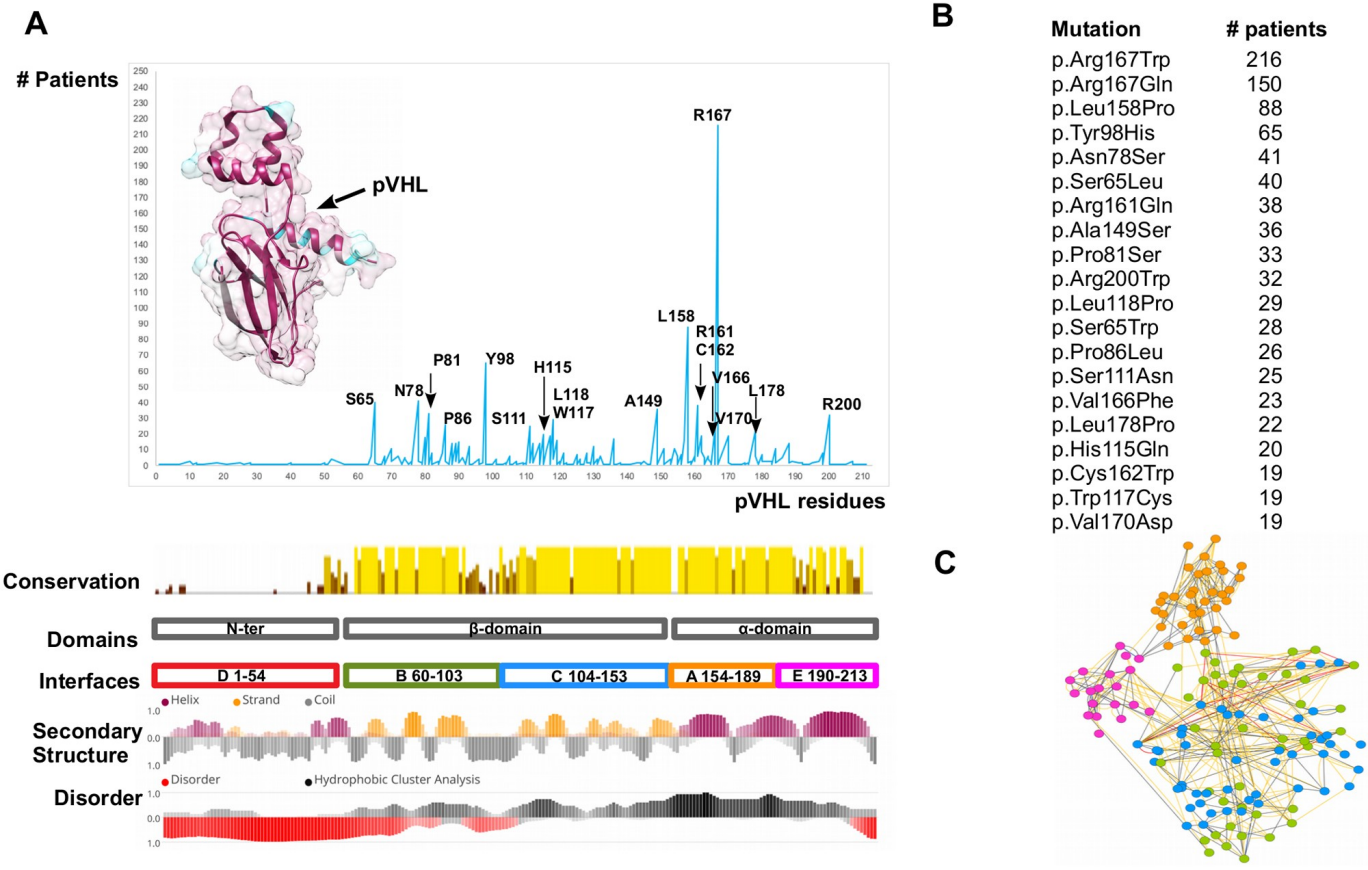
The most frequently mutated pVHL positions. (A) pVHL mutations are grouped and collected based on their occurrence among VHL patients. The pVHL 3D structure is presented as cartoon and colored by sequence conservation. Interfaces definition, secondary structure and disorder content are presented below. (B) The most frequent pVHL mutations. (C) RING representation of pVHL residues interaction network, colored by secondary structure. Edges represent connection among residues (nodes), with red representing π-π stack interaction, blue salt bridges while yellow and grey for van der Waals and hydrogen bonds respectively.

**Fig 2:**
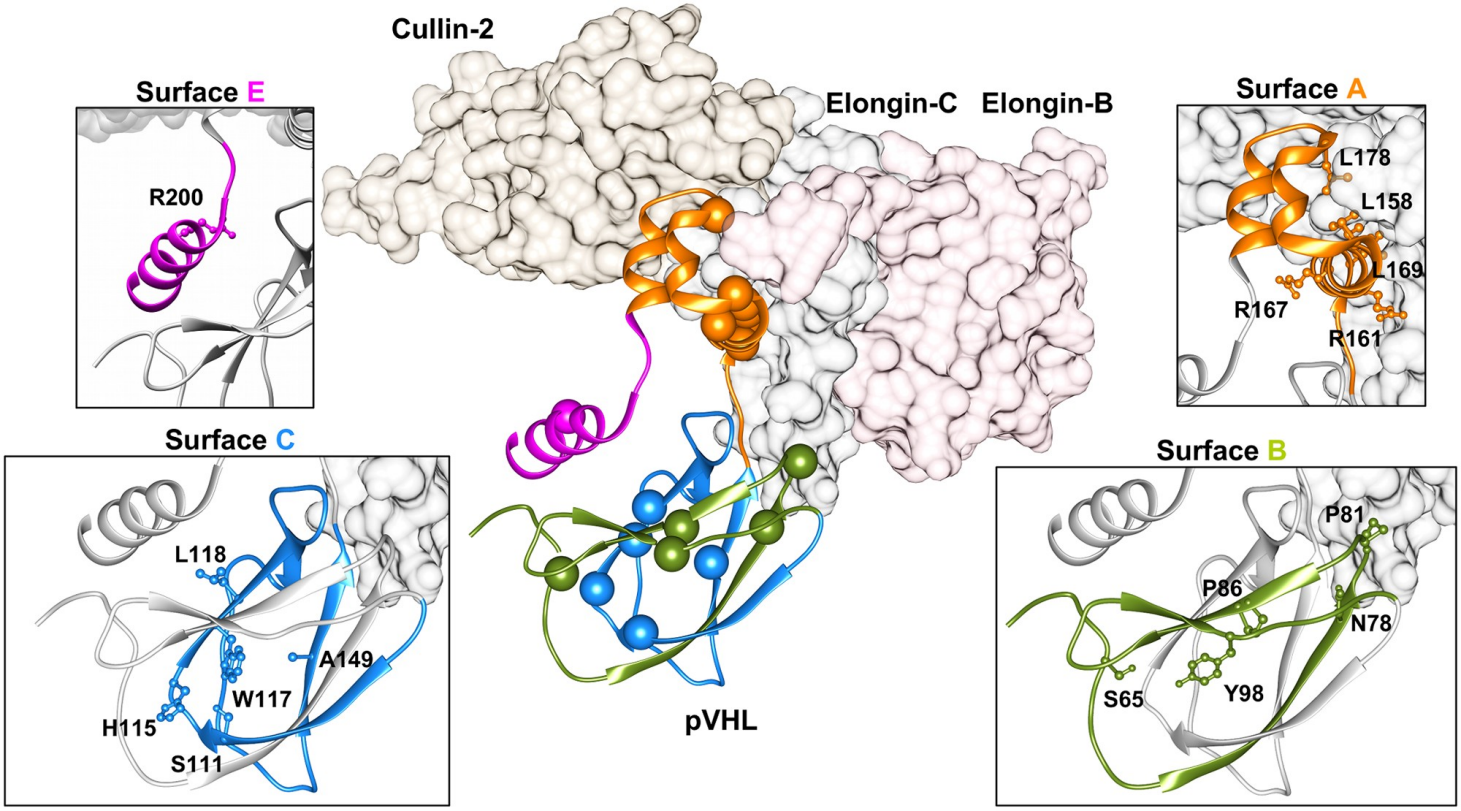
Overview of the VCB complex. Elongin-B and -C and Cullin2 are represented as plain surface and colored in pink, dark grey and brown, respectively. Cartoon represents pVHL, with the most mutated residues presented as spheres. Zooms show different pVHL binding interfaces colored according residues forming the interface. In particular, orange is for surface A, while green and blue are for surfaces B and C respectively. C-terminal region (surface E) is presented in magenta.

A consensus approach based on multiple pathogenicity and stability predictors was used to systematically explore the phenotypic impact of pVHL mutations. Mutations localizing at the N-terminal tail are mostly ranked as benign, while those affecting both alpha- and beta-domains are predicted to damage protein structure or reduce its stability. Interestingly, two synonymous mutations are also predicted as probably damaging (S1 Table). As pVHL mutations appear to indiscriminately affect the entire protein surface, we wondered whether some mutations were more frequent in patients. We calculated the number of mutation sites found above the 95^th^ quantile of the mutation dataset and identified 18 frequently mutated pVHL positions (Fig 1). Most residues present multiple amino acids substitutions, e.g. 7 different variants are described for Asn78, and localize in both alpha and beta pVHL domains (Fig 1 and S2 Table). Moreover, all associate with malignant RCC paired with multiple VHL manifestations. Residue-residue interaction network built from pVHL 3D-structure suggests that these mutations disrupt multiple interactions (S3 Table). In particular, Arg167 is the most frequently mutated pVHL residue (366 patients), with the two main variants p.Arg167Trp and p.Arg167Gln. This residue localizes on surface A, which is involved in VCB complex formation. Inspection of complexes formed by pVHL, Elongin-B and -C and cullin-2 with RING [33] shows the residue to be located on the inner side of pVHL α-helix 1, directly interacting with Elongin-C (Fig 2). Arg167 forms electrostatic interactions with Glu160 and Asp126, playing a structural role in maintaining the correct α-domain fold. Intriguingly, the phenotypes described for the two variants are slightly different. Both associate with RCC, pheochromocytoma, hemangioblastoma, paraganglioma and cyst formation. Patients harboring p.Asp167Trp are also found to develop epididymal/ovarian cystadenoma (1 occurrence), while presence of endolymphatic sac tumor is reported only for p.Asp167Gln (2 occurrences). Despite the exiguous number of reported patients, this finding suggests that the two changes may support different tissue-specific pVHL functional impairment. The p.Arg161Gln mutation destabilizes the interaction between pVHL and Elongin-C. This arginine forms a salt-bridge with Elongin-C Glu92 and substitution with glutamine impairs this interaction, weakening VCB complex formation. Collectively, our analysis shows the most frequent mutations affecting surface A to exert pathogenicity by directly impairing VCB complex formation rather than compromising the pVHL/HIF-1α interaction. Conversely, mutation p.Tyr98His on surface B exerts its pathogenic effect by directly disrupting pVHL association with HIF-1α. Tyr98 plays a predominant role in pVHL substrate recognition, forming a hydrogen bond with the HIF1-α Pro564 backbone. Of note, this specific HIF1-α proline is crucial for pVHL interaction upon hydroxylation [10]. Other frequent mutations localizing on surface B (p.Ser65Leu, p.Asn78Ser, Pro81Ser) are predicted to play a role in destabilizing the β-domain. Structural investigation shows these residues mostly face the inner layer of the β-domain and are not directly in contact with conventional pVHL interactors, i.e. Elongin-B and -C, at least based on the currently available crystal structures. Similarly, mutation p.Ala149Ser affects surface C and is localized on the seventh strand of the β-domain. Ala149 contributes to the pVHL hydrophobic core, forming van der Waals interactions with Val74, Phe76, Phe119, Val130 and Phe136. Substitution with serine may drive pVHL fold destabilization and VHL syndrome insurgence. Finally, p.Arg200Trp is the only frequent mutation in the pVHL C-terminal tail and mostly associated to polycythemia. It disrupts a salt bridge between Arg200 and Glu134 which helps the α-domain to fold correctly.

### Protein networks impaired by the most frequent pVHL mutations

**Fig 3:**
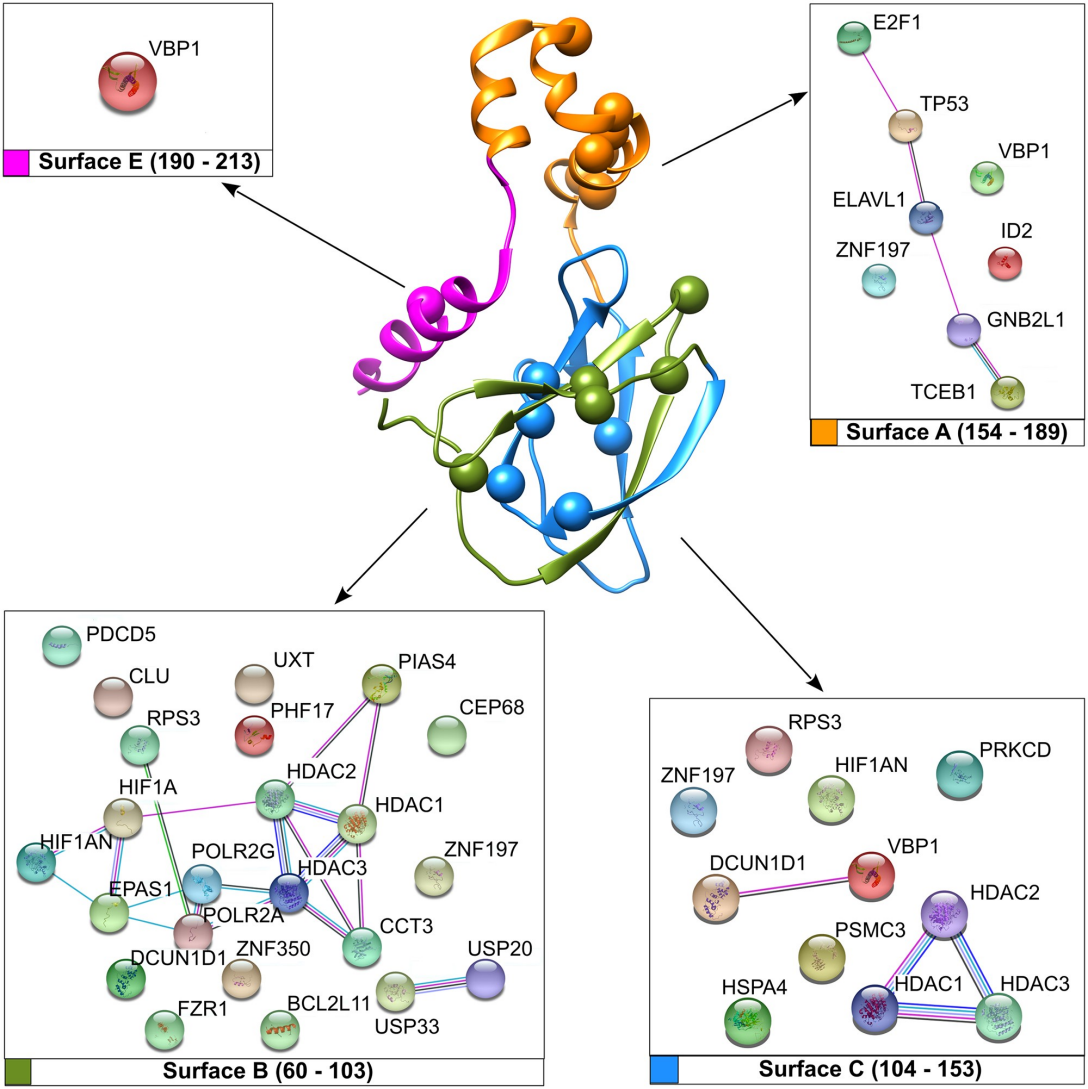
Network of pVHL interacting proteins and associated pVHL surfaces. Manually curated pVHL interactors from VHLdb are represented as colored sphere with connecting edges representing physical interaction among them.

Our preliminary mapping of the most recurring pVHL mutations provides some useful hints about their pathogenic effect. Such an interpretation however fails to explain the variability of phenotypes observed in VHL patients (S4 Table). A possible interpretation is that these amino acid changes disrupt pVHL association with interactors other than Elongin-C, Elongin-B and HIF-1α. To address this issue, we extracted from VHLdb interactors known to specifically bind pVHL regions corresponding to the most frequent mutations (S5 Table). These were used to generate protein association networks describing the putatively compromised cellular processes (Fig 3). The surface A interaction network collects eight proteins mainly involved in DNA/RNA processing. pVHL loss is frequently associated to genomic instability in renal cancer [34]. In particular, pVHL null cells display reduced activation of p53-mediated apoptotic response [35], as well as abnormal cell-cycle arrest upon DNA damage, with normal response observed after pVHL restoration. We searched for highly interconnected regions of the network around surface A using MCODE [36] to better characterize these observations. Three main clusters representing macromolecular protein complexes were found (S1 File). The first cluster is formed by five proteins belonging to the prefoldin protein family, a group of chaperon forming folding complexes [37]. As pVHL is thought to interact with more than four-hundred different interactors [29], different prefoldin proteins may assist formation of multiple pVHL-driven protein complexes. Surface A mutations may interfere with complex assembly, promoting abnormal cell behavior. The second cluster accounts for proteins directly involved in p53 activation, supporting a pVHL role in this specific cellular function. The last cluster for surface A includes Elongin-C and Elongin-B, confirming the role of this interface in VCB complex assembly. Of note, this cluster also includes SOCS3 (Suppressor of cytokine signaling 3), a protein acting as negative regulator of cytokine signal transduction. SOCS3 binds pVHL to form a heterodimeric E3 ligase complex targeting the JAK2 kinase for degradation [38]. This interaction was proposed to play a role in Chuvash polycythemia insurgence [38], a familiar polycythemia form caused by pVHL mutations. Collectively, our findings suggest that interface A is involved in formation of multiple protein complexes. Mutations affecting this region may result in abnormal gene transcription as well as deregulation of apoptotic response and signaling. Mutations on surface B can compromise pVHL association with 23 different interactors. Surface B contains the HIF-1α binding site and is also the binding interface of multiple proteins involved in regulating the pVHL E3-ligase activity. The two de-ubiquitinating enzymes USP33 and USP20 are clear examples. This specific enzyme class is involved in regulating multiple pathways by modulating protein degradation. USP33 and USP20 in particular play a relevant role in beta-adrenergic receptor (ADRB2) homeostasis. Upon prolonged agonist stimulation, they constitutively bind ADRB2 and inhibit its lysosomal trafficking [39]. The pVHL interaction with at least three different histone deacetylases was also found putatively compromised. In addition to being an E3 ligase component, pVHL binds histone deacetylases to form a heterodimeric complex, acting as a transcriptional co-repressor to inhibit the HIF-1α trans-activation function [40]. Unexpectedly, our network analysis of surface B interactors shows only one relevant cluster accounting for ten ribosomal proteins. Recently, pVHL was proposed to inhibit both ribosome biogenesis and protein synthesis by inducing nuclear retention of pre-40S ribosomal subunits [41]. The biological meaning of this interaction is however far from understood. Taken together, our findings suggest surface B to be mainly involved in protein degradation pathways. Based on these data, we suggest that the pathogenic assessment of mutations affecting this area should also include other putative pVHL hydroxylated-substrates beyond the sole HIF-1/2α, e.g. SPRY2 [42], ADRB2 [43], EPOR [44]. Eleven interactors are found to be affected by surface C mutations. Cluster analysis suggests this interface also to play a role in ribosome biogenesis and protein synthesis, as already observed for surface B. We found a second cluster collecting members of the histone-deacetylation protein family, i.e. HDAC1-3. This suggests interface C to also have an important role in transcriptional regulation and cell cycle progression. Finally, a single cluster collecting proteins involved in matrix organization, ciliogenesis and BBsome assembly (Bardet-Biedl syndrome, an octameric protein complex required for ciliogenesis and centriolar function [45]) was found to specifically interact with the pVHL C-terminus, suggesting this region may form a further pVHL interaction interface (S1 File).

### Distribution of pVHL mutations and associated phenotypes

**Fig 4:**
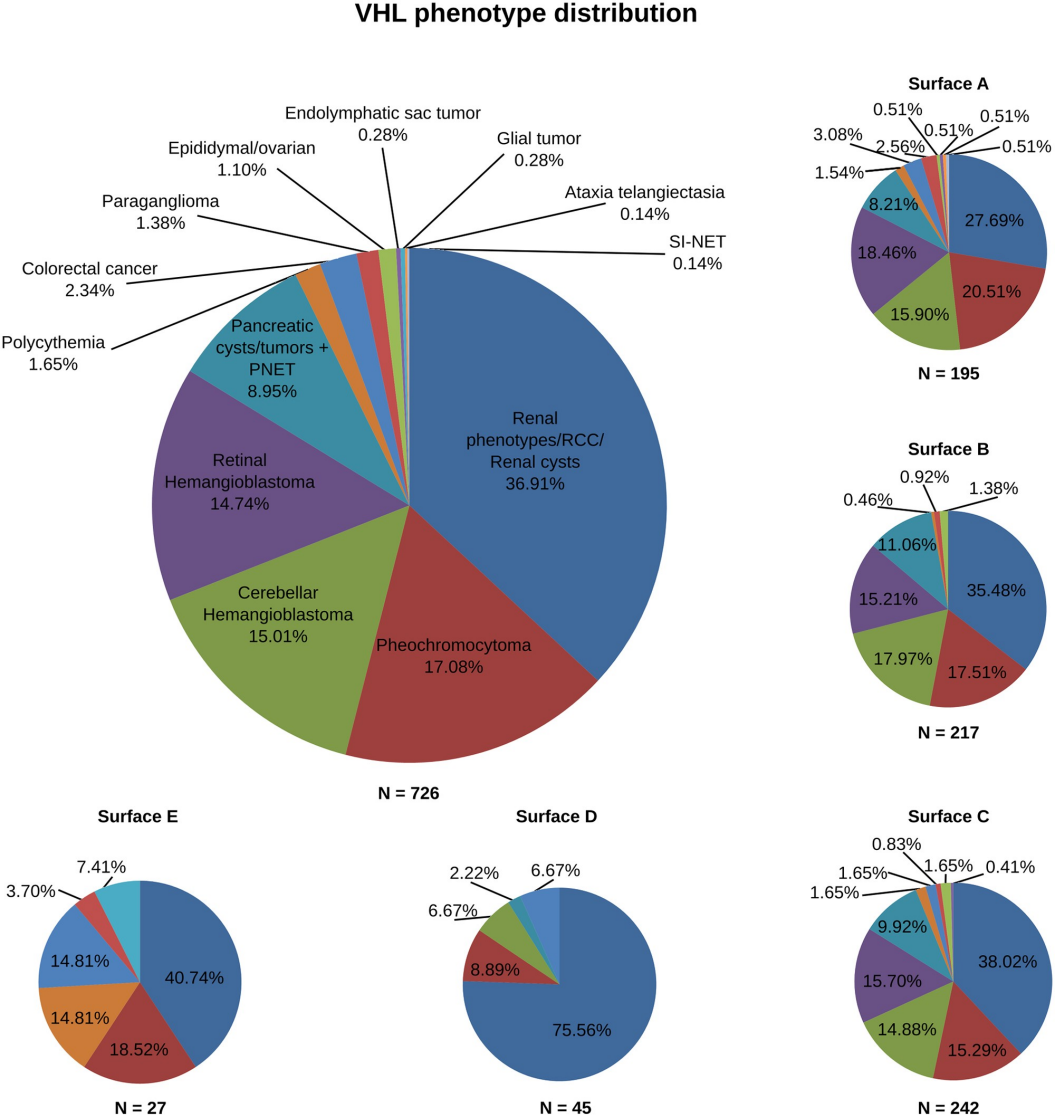
Distribution analysis of pVHL mutations and related phenotypes. The large circle shows the percentage of mutations for which a specific phenotype is reported from patients. The same distribution is reported for each pVHL surface (small circles). HB = hemangioblastoma; PNET = pancreatic neuroendocrine tumor; RCC = renal cell carcinoma; SI-NET = small intestine neuroendocrine tumor.

The current classification describes two main types of VHL disease based on their propensity to develop pheochromocytoma [16], Type 1 (low risk) and 2 (high risk), respectively. Three sub-types are further proposed for Type 2 to appraise the risk of developing renal carcinomas. These differences can be interpreted as resulting from impairment of different protein-protein interactions. We wondered whether the position of mutations on pVHL surfaces may associate with specific disease manifestations. We retrieved 1,670 mutations affecting different pVHL binding interfaces [27,29] and isolated 742 amino acid variations associated to VHL phenotype, e.g. only substitutions described as yielding RCC (S6 Table). We focus on mutations affecting single residues on each binding surface, to isolate pVHL areas that can support unknown functions or binding motifs. Unsurprisingly, our analysis shows mutations to distribute over the entire protein surface. However, differences in localization were observed (Fig 4). Normalizing the number of mutated positions over the number of residues forming each interface shows surface B to present the highest number of mutated positions (39/43), indicating that 91% of residues forming this interface are targeted by VHL mutations. Surface C (43/49; 88%) and surface A (28/35; 80%) have slightly fewer mutations, followed by the C-terminal tail (15/23; 65%) and interface D (27/59; 56%). Surfaces B and C form together the β-domain of pVHL, which includes the HIF-1α binding site. These preliminary findings can be easily explained accounting for the specific domain function. Surface A includes the pVHL α-domain needed to sustain interaction with Elongin-B and -C to form the VCB complex. The meaning of mutations affecting both surface D and the protein C-terminus is more difficult to address. Surface D is an accessory acidic tail present only in the pVHL30 isoform [46]. A pVHL30-specific role in regulating p14ARF tumor-suppressor activity has been proposed [47], however this function is currently debated. We investigated whether phenotypes associated with these mutations can be used to draw surface/phenotype correlations. Our analysis shows that the substitution of residues forming interfaces B and C yields similar phenotypes (Fig 4). Renal disease, globally including ccRCC, RCC and renal cysts, is on average the main manifestation associated with these two interfaces. A similar tendency is also observed for hemangioblastoma (both cerebellar- and retinal- forms) and pancreatic lesions (cysts and tumors). Pheochromocytoma is slightly more frequent when mutations affect surface B (17.5%) than surface C (15.3%). Impairment of surface C is also associated to other minor phenotypes which are virtually absent in surface B, e.g. colorectal cancer and endolymphatic sac tumor. In the same way, surface A mutations are associated with almost the entire spectrum of VHL phenotypes (Fig 4). Renal disease (27.7%), pheochromocytoma (20.5%) and hemangioblastoma (34.4%) are similarly distributed. Minor manifestations are also present and uniformly distributed, even though accounting for smaller numbers (< 3.5%). Conversely, mutations affecting surface D and the C-terminal tail describe a different scenario. Surface D mutations, which are only present in the pVHL30 isoform, are mostly associated with renal disease (75.6%). Of note, cerebellar-hemangioblastoma (6.7%) is the only hemangioblastoma subtype described for mutations affecting this interface. This suggests that pVHL30 may play a cerebellar-specific role which has no equivalent in retinal tissue. Finally, C-terminal mutations are mainly characterized by renal syndrome (40.7%) and pheochromocytoma (18.5%). Intriguingly, mutations associated to non-canonical VHL manifestations, such as polycythemia (14.8%), colorectal cancer (14.8%) and glial tumor (7.4%) seem mostly to derive from mutations of this interface. Structural investigation suggests that changes in this region should neither impair HIF-1α nor VCB complex formation. These findings collectively suggest that the C-terminal region may play a functional role in other HIF-independent pVHL functions. The analysis confirms that the three main interfaces (A-C) present no statistically significant difference in tumor type association. Conversely, the different correlations observed for the two pVHL tails are statistically significant (p-values <0.05). Analyzing the data presented so far from a disease point of view, we observe that renal disease, pheochromocytoma and pancreatic insults derive from mutations affecting all pVHL interfaces. Similarly, pheochromocytoma behavior suggests that both phenotypes are associated with general pVHL impairment. Instead, retinal-hemangioblastoma arises from mutations limited to the three main interfaces (A-C). The cerebellar subtype also seems to include mutations on interface D present only in the pVHL30 isoform. It will be very interesting to investigate whether these differences can be explained with a functional specialization of pVHL30 in cerebellar tissues.

### pVHL interactors and disease states

VHL disease is characterized by slow progression coupled to a plethora of different symptoms. Detailed molecular data for 59 pVHL interactors involves pVHL surfaces reported to engage in multiple protein-protein interactions [29,47,48]. We selected VHLdb single point mutations reported to associate with a single VHL manifestation (S6 Table) to suggest binding interfaces correlated with specific pVHL functions. Unsurprisingly, we observed that many mutations included in this reduced subset affect regions involved in protein-protein complex formation (S7 Table). This finding suggests a single amino acid substitution can impair formation of multiple associations. On the other hand, it makes retrieving which specific interaction is actually weakened or damaged by the mutation more difficult. Considered these data, we decided to isolate only pVHL interactors unaffected by the mutation associated to a specific phenotype to reduce incoherence and lower the risk of redundancy, generating a set of negative snapshots describing pVHL interactions and pathways not directly compromised by a specific mutation (Table 1). Our data show that mutations promoting RCC, and more in general renal disease, can interest virtually all considered interactors. This is coherent with an almost complete functional inactivation of pVHL, in particular for those connected with hypoxia response. Similarly, mutations only associated with pheochromocytoma severely affect the DGKZ, PRKCI, PRKCD and PRKCZ kinase binding interfaces, further suggesting a link between hypoxia sensing and phosphorylation-mediated signaling. Mutations associated only to this neuroendocrine tumor affect the pVHL association with almost the entire subset of interactors considered. Notably, associations with AKT1 and HSPA4 are not impaired in pheochromocytoma. AKT1 is known to promote survival and proliferation of various cancers [49]. Its interaction with pVHL is proline-hydroxylation-dependent and yields complete inhibition of AKT1 activity [50]. Our finding indicates that cells harboring these mutations may retain AKT1 inhibition and suggest that proliferative pathways modulated by pVHL/AKT1 association are not compromised. The retained HSPA4 interaction seems to go in exactly the same direction. HSPA4, also known as HSP70RY [51], is a member of the HSP70 chaperone family proposed to rescue premature degradation of pVHL mutants, inducing their stabilization and halting tumor progression [52]. Collectively, these data are in agreement with the slow progression rate reported for pheochromocytomas [53] and describe this cancer to arise only from partial pVHL inactivation. Mutations associated with both cerebellar- and retinal-hemangioblastoma localize in regions not affecting the pVHL interaction with transcription factors (TP53, ELAVL1) and regulators (ID2, E2F1, ELOC). Although pVHL is known to interact with two RNA polymerase II subunits, RBP1 [54] and RBP7 [55], its role in regulating gene transcription is debated [28]. According to our data, cell damage promoting hemangioblastoma should be ascribed to inactivation of HIF-dependent functions. Conversely, pVHL mutations associated to colorectal cancer do not affect association with HIF-1α, TP53, or proteins involved in extracellular matrix (ECM) formation and turnover, such as TUBB, TUBA1A, TUBA4A, KIF3A and KIF3B. Interestingly, a single mutation is observed as never inducing both paraganglioma, a tumor originating from paraganglia in chromaffin-negative glomus cells, and cystadenoma, a tumor of epithelial tissue with glandular origin. Gene ontology terms analysis of pVHL interactors shows enrichments in pathways mediating transport along microtubule, modulation of protein kinase C activity and microtubule organization (S1 Fig), predicting that pVHL alterations are not limited to hypoxia signaling. Similarly, enrichment generated from disease-dedicated databases shows pVHL to putatively play a role also in other malignancies normally not associated to VHL syndrome. Gene networks of pVHL interactors built taking under consideration tissues developing cancer in VHL syndrome highlight an almost conserved core interaction network (S2 Fig). Indeed, we found some similarity between networks derived from central nervous, kidney and pancreatic tissues, in which pVHL interactors connected with the cytoskeleton stability and histones deacetylation show a significant relationship coefficient. Vice versa, no marked conservation was found for retina and adrenal gland tissues suggesting that malignancies developing in these body districts may require different pathway alterations.

**Table 1:** List of interactors putatively not affected by pVHL mutations found in patients presenting a specific a VHL phenotype (*left*). Phosphorylation sites found to be frequently mutated in both renal manifestations of VHL (collectively including RCC, ccRCC, renal cysts) and pheochromocytoma (*right*).

Conserved interactions		Mutated phosphorylation sites
Pheochromocytoma	Hemangioblastoma	Renal disease & Pheochromocytoma
HSPA4	TP53	AURKA
AKT1	ID2	CSNK2A1
	ELAVL1	CHEK2
	E2F1	GSK3B
	ELOC	NEDD8

### Post translational modifications of pVHL

Considered that several phosphorylation sites appeared to be mutated in tumor samples from patients, we wondered whether alteration of other post translational modification (PTM) sites may play a role in VHL progression. We found multiple missense mutations overlapping a total of 14 PTM sites distributed on all of the pVHL binding surfaces (S8 Table, S3 Fig). Three residues localized in the surface D, Ser33, Ser38 and Ser43 are phosphorylated by CSNK2A1, which is known to reduce pVHL stability, as well as to affect HIF-1α and P53-mediated transcription [56–58]. In the contest of VHL syndrome, we found the Ser33 and Ser38 to be mutated in RCC and pheochromocytoma. We also found mutations in sites recognized by AURKA, CSNK2A1, CHEK2, GSK3B. The latter in particular, is involved in the regulation of microtubule dynamics [57,59] and phosphorylates Ser68. Mutation of this residue associates with RCC, retinal hemangioblastomas and pheochromocytoma. Surface B is also phosphorylated by AURKA [60] and CSNK1A1 [57,59] at Ser72, and patients with mutations affecting this residue develop hemangioblastomas and RCC. Phosphorylation of pVHL by AURKA is thought to modulate microtubules stability and dynamics [60], while CSNK1A1 is involved in the reorganization of cytoskeleton [57,59]. Surface C is modified at Ser111 by CHEK2 [61], whose missense substitution associates to retinal- and CNS-hemangioblastomas, pancreatic and renal cysts, RCC and pheochromocytoma. We found seven NEK1-targeted phosphorylation sites localizing onto surface B and C. Five of them are found to be mutated in patients developing all of the major phenotypes characterizing the VHL syndrome. NEK1 phosphorylates pVHL to promote its proteasomal degradation and ciliary destabilization [62]. Other pVHL PTM mutated sites include neddylation upon NEDD8 interaction of Lys159 (RCC and pheochromocytoma), which is required for fibronectin matrix assembly and suppression of tumor development [63] and sumoylation of Lys171 by PIASy [64,65], found mutated in RCC. Two surface B residues, Arg79 and Arg82, are methylated by the protein arginine methyltransferases family (PRMTs) [62]. They are found to be mutated in different VHL phenotypes including RCC, polycythemia, and pancreatic tumors, however the exact biological meaning of this pVHL modification is not yet characterized. Ubiquitination of pVHL is known to occur at residues Lys155, Lys171 and Lys196 [65–67]. According to our data, we found only Lys171 to be mutated in RCC. Collectively taken, these results suggest that several VHL pathogenic variants can exert their effect by disrupting and/or altering complex signaling networks regulated by proteins involved in PTM events.

### Mutations promoting polycythemia and other interesting cases

**Fig 5:**
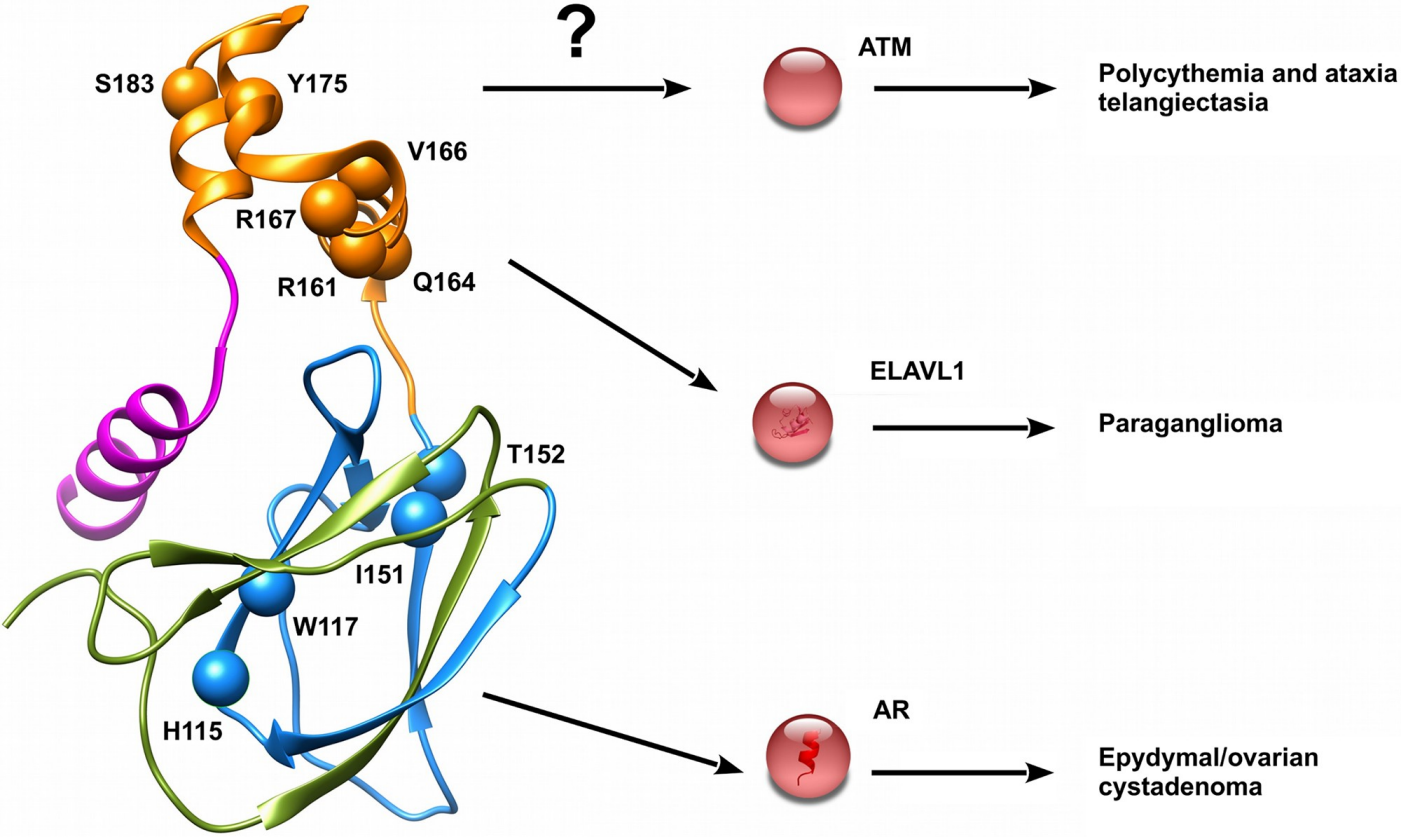
Mutations of pVHL associated to specific VHL phenotypes. Mutations promoting interesting VHL phenotypes are presented as red spheres. Interactors putatively affected and other less frequent phenotypes are reported on right.

Although a single mutation is never associated to either paraganglioma and cystadenoma as the sole phenotype observed in patients, we found specific mutations which can improve our knowledge on their onset. The five mutations p.Arg161Gln, p.Gln164His, p.Val166Phe, Arg167Trp and Arg167Gln localize in a small area forming the ELAVL1 binding interface (Fig 5). Increased expression of this protein was recently proposed [68] to be linked with the metastatic potential of both paraganglioma and pheochromocytoma. Similarly, the three mutations p.His115Arg, p.Trp117Gly and p.Ile151Phe localize on the androgen receptor (AR) binding interface, suggesting that these specific interactors may play a crucial role in cystadenoma insurgence (Fig 5). We also found p.Tyr175Cys linked to the insurgence of polycythemia associated with ataxia telangiectasia (AT) [69]. This is a rare recessive disorder characterized by progressive cerebellar ataxia, dilatation of blood vessels in conjunctiva and eyeballs, immunodeficiency, growth retardation and sexual immaturity. Interestingly, AT is caused by mutations affecting serine-protein kinase ATM (ataxia telangiectasia mutated), a pVHL interactor [18] of unknown binding interface.

### Predicting pVHL association with the most promising interactors

**Fig 6:**
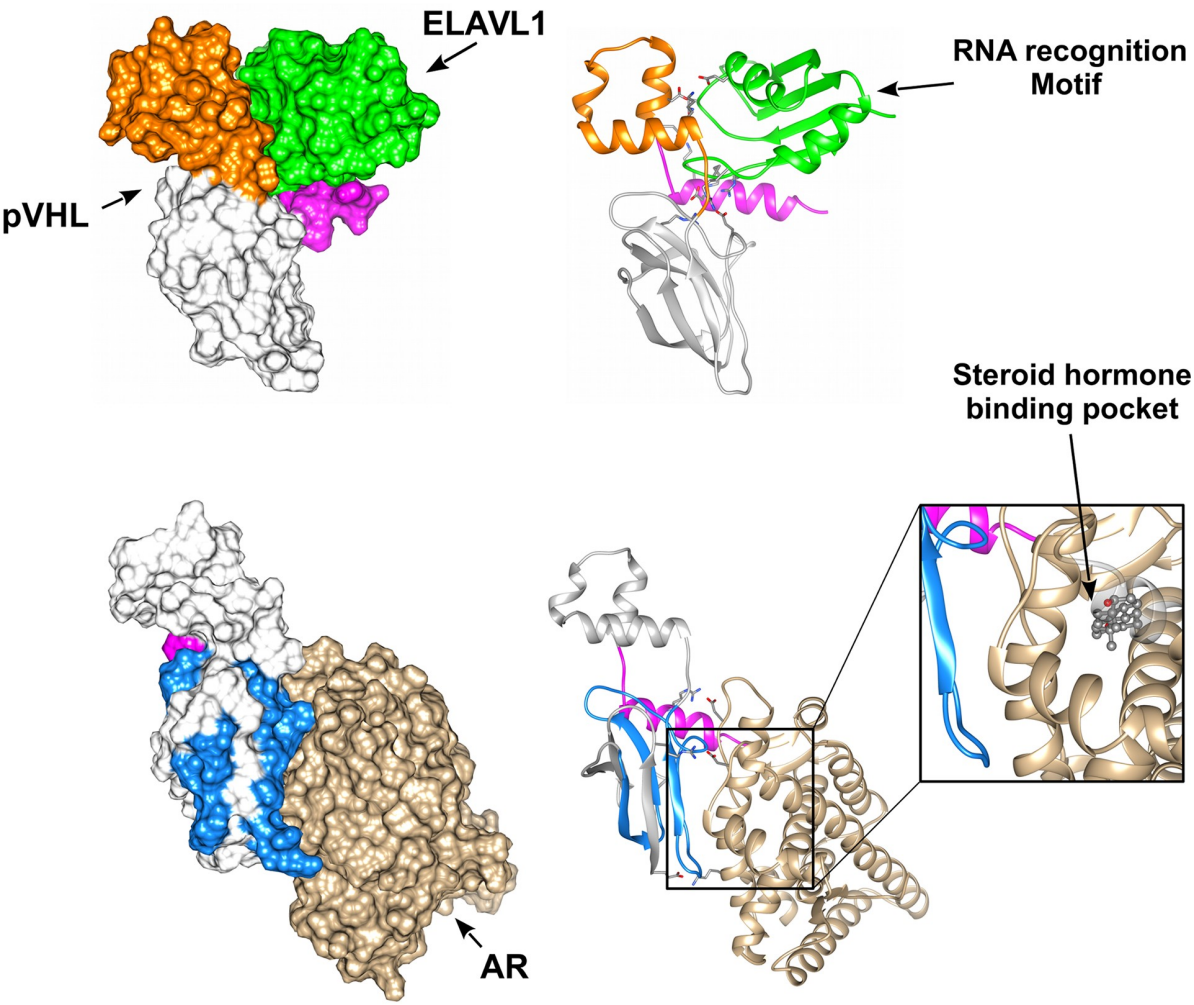
Putative pVHL molecular complexes with ELAVL1 and AR. The best scoring complexes are presented and colored according pVHL interface drawing the interaction. ELAVL1 is presented in green while brown is for AR. Zoom shows the AR ligand-binding pocket predicted to be impaired upon pVHL interaction.

We wondered whether the binding interface data may be used to perform protein docking studies of pVHL with its multiple binding partners. We modeled the pVHL interaction with ELAVL1 and AR. Association with ELAVL1 is sustained by the pVHL α-domain and the RRM1 domain of ELAVL1 [70]. As the pVHL α-domain is relevant for VCB complex formation, we first searched for possible structural analogy to perform docking by homology, but no suitable template was found. Multiple models are generated and ranked through concordance with literature data. The best docking model predicts this interaction to involve both pVHL interface A and the C-terminal tail (Fig 6). Inspection of the interacting residues and electrostatic surface analysis shows this association to involve several electrostatic interactions. A mostly negatively charged pVHL pocket accounts for 7.2% of the pVHL accessible surface interacting with an ELAVL1 area of opposite charge (S4 Fig). Based on this model, the five mutations p.Arg161Gln, p.Gln164His, p.Val166Phe, Arg167Trp and Arg167Gln are not directly involved in ELAVL1 binding, but rather allow the right positioning of helices forming the pVHL α-domain.

The docking model of the pVHL-AR complex shows pVHL surface C interacting with α-helices H3 and H11 (Fig 6) of the AR ligand-binding pocket [71]. This justifies the inhibitory effect, as pVHL mutations p.His115Arg, p.Trp117Gly and p.Ile151Phe are predicted to disturb association with AR and directly interfering with correct folding of interface C. We observed that both pVHL interactions with ELAVL1 and AR are predicted as mainly electrostatic. This is coherent with the comparatively high number of exposed charged residues present in pVHL, i.e. 27.2% positive and 14.7% negative residues. On the other hand, these findings also suggest that small clusters of pVHL mutations can disturb the binding of entire pVHL interfaces by promoting local unfolding and disturbing a specific binding area. This mechanism may be easily used by cancer cells to inactivate an entire set of pVHL functions by single mutation, rapidly acquiring a fitness advantage over neighboring healthy cells.

### Simulating pVHL pathway impairments

To better clarify how cell modifications due to differentially impaired pVHL interactors prompt tumor transformation, we simulated the most promising impaired associations using a Petri net description of the pVHL interaction network [32]. Considering the interesting findings from the docking analysis, we simulated the impaired associations of pVHL with ELAVL1 and AR. Interaction of pVHL with ELAVL1 is thought to play a role in p53 expression and stabilization [72]. Loss of this interaction is predicted by our model to induce enhanced VEGF production and stabilization through NR4A1 [73] (Nuclear receptor subfamily 4 group A member 1) mediated inhibition of HIF-1α degradation. In parallel, we registered a glucidic metabolism imbalance due to overproduction of pro-opiomelanocortin (POMC), the precursor of the proopiomelanocortin hormone. We also observed deregulation of the Krebs cycle paired with partial PHD2 inhibition from sub-products of the carbohydrate metabolism. Alterations of proopiomelanocortin levels are a common trait of VHL-related tumors [74,75]. These findings show that several mutations potentially impairing the interaction of pVHL with ELAVL1 may induce severe cell adaptations promoting tumor transformation.

AR is a steroid hormone receptor playing a pivotal role in cancer through androgen-induced gene transcription and kinase-signaling cascade activation [76,77]. pVHL is thought to suppress its activity, inhibiting both genomic and nongenomic AR functions [78]. Loss of pVHL-mediated AR inhibition is predicted as hyper-activating MAPK/ERK (mitogen-activated protein kinase) signaling. Under physiological conditions, MAPK/ERK activation is also stimulated by VEGF [79]. We registered enhanced vascular growth and augmented vascular permeability mimicking improved VEGF production. In parallel, we observed an increased metabolic activity sustained by the large oxygen availability generated from new vessels formation. Intriguingly, proliferative pathways typical of malignant progression are predicted to be inactivated, in particular, by JADE-1 tumor-suppressive functions through induction of apoptosis [80] and Wnt signaling inhibition [81]. Our model suggests a benignant phenotype characterized by high vascularization but reduced proliferation. This behavior is in agreement with the cystadenoma insurgence observed in patients harboring pVHL mutations localizing on the AR binding interface (p.His115Arg, p.Trp117Gly and p.Ile151Phe).

We also wondered whether this approach can be used to simulate loss of pVHL interaction with different kinases. We selected GSK3β and ATK-1, as representatives of phosphorylation-mediated pVHL modulation and pVHL-dependent kinase inhibition respectively. GSK3β is known to phosphorylate pVHL at Ser72 [59]. This modification is linked with micro-tubule instability and external matrix remodeling. Our model predicts that upon impairment of GSK3β/pVHL association, glycogen metabolism is also impaired as enzymes deputed to glycogen synthesis are hyper-activated. Enhanced GSK3β-mediated phosphorylation of HIF-1α and its concurrent pVHL-independent degradation [82] are also predicted. In a real cell, these findings can be seen as activation of a cellular reserve mechanism for adapting its function in response to tumor suppressor mutations. GSK3β is inhibited upon insulin stimulation [83], suggesting that cells harboring mutations affecting GSK3β/pVHL association may undergo relevant HIF-1α functional deregulation when exposed to concurrent hypoxia and insulin stimuli. Finally, as pVHL is thought to inhibit AKT1, loss of pVHL association with AKT1 is predicted to promote AKT1 hyper-activation [50], which in turn promotes activation of cell survival pathways, microtubules glycolysis as well as external matrix destabilization. In a cellular environment, these modifications can collectively be interpreted as enhanced anchorage-independent growth and tumor transformation.

## Discussion

We investigated the effect of mutations affecting the pVHL tumor suppressor and their correlation with different phenotypes described for VHL patients. Our efforts in deciphering pVHL functions arise from the consideration that clinical manifestations of this familiar predisposition to develop cancers may vary among patients harboring the same mutation. The best known pVHL function is its role in degrading the HIF-1α transcription factor [12]. Its role as hypoxia sensing component was conveniently used to explain some of the main VHL manifestations. However, it fails almost entirely in predicting the pathogenic risk of several mutations not directly connected with HIF-1α degradation. A robust body of literature is prompting that pVHL possesses other HIF-independent functions which co-participate in explaining the difference in disease progression clinically observed in patients [17,6,84]. We previously presented VHLdb, a database collecting interactors and mutations of the human pVHL [29] aimed at rationalizing the existing knowledge around pVHL. This data is used here as a starting point to shed light on VHL disease. As a first result, we found that the most frequent mutations impair either the pVHL role in VCB complex assembly or promote β-domain destabilization. Unsurprisingly, these findings confirm that the most relevant pathogenic effect related to VHL insurgence is an inactivation of the E3-ligase function. However, an analysis of pVHL interactors putatively affected by these same mutations tells a more complex story. Our investigations hint at the possibility of associating a specific function to each pVHL surface. Based on our findings, we propose interfaces A and C of pVHL to be mainly associated with proteins involved in gene transcription and regulation and interface B to regulate protein homeostasis of several pVHL interactors. A novel C-terminal interface addresses ECM and ciliogenesis associated pVHL functions. The difference in binding property may also reflect different contributions to disease manifestations. Not all VHL patients develop the same phenotypes, in particular hemangioblastoma and renal disease and are found to be the predominant manifestations. Renal disease appears to be equally represented by mutations affecting the three main pVHL interfaces, while it is the predominant phenotype described for mutations localizing on surface D. This interface is formed by a long intrinsically disordered tail present only in the pVHL30 isoform, suggesting this specific isoform to play a precise role in renal cancer insurgence. Both paraganglioma and cystadenoma were never found as single VHL phenotypes, suggesting that these two tumors arise as secondary manifestations of VHL, possibly pairing pVHL impairment with the functional inactivation of other relevant players. We also found several mutations affecting pVHL PTM sites, indicating that malignant phenotypes can arise from pVHL functional deregulation rather than structural disruption. In particular, our simulation of phosphorylation events impaired by cancer-related mutations shows that correct interpretation of VHL fate benefits from the integration of different information sources. Manual curation and interpretation of literature data can represent a powerful tool to decipher the molecular role of this tumor suppressor protein. Collectively taken, our findings provide direct biological insights into VHL-associated tumors and may help designing novel experimental investigations to elucidate novel treatment paradigms for VHL syndrome.

## Methods

A schematic representation of the analysis workflow is presented in S5 Fig, while detailed information as following.

### pVHL mutations analysis

Germline and somatic pVHL mutations were retrieved from VHLdb [29] and enriched with manual curation the most relevant literature published from 1994 to 2018 (S9 Table). Pathogenicity and stability prediction were assessed with PMut [85], Polyphen-2 [86], Panther [87] and Align-GVGD [88]. Statistical analysis was performed with R software (http://www.R-project.org). In particular all mutations accounting for a number of patients greater than the 95th quantile (p = 0.05), computed considering the vector of patient numbers, were selected for further analysis. Renal VHL manifestations (i.e. ccRCC, cysts) were collectively presented as renal disease due to many case report papers describing novel VHL mutations lack a precise typing of cancer sub-type, e.g. generically referring to either benign renal cancer or renal carcinoma. The pVHL surfaces were defined as previously described [27,29]. Missense mutations were mapped and their positions compared using Chimera [89] on three different pVHL 3D-structures representing the VCB complex (PDB codes: 1LM8, 1LQB, 4WQO). Difference in mutation distribution for each surface was evaluated by ANOVA test (p = 0.05). Networks of interacting residues affected by mutations were calculated with RING 2.0 [33]. Variation of pVHL sequence features upon mutations was calculated with FELLS [90].

### pVHL interactors analysis

Manually curated pVHL interactors with known pVHL binding surface were retrieved from VHLdb [29] (high confidence dataset). The protein interaction network around each pVHL interface was built with Cytoscape [91] and extended with a second shell of STRING [92] interactors (< = 10 interactors, confidence > = 0.700, no text mining). Interactors were clustered and investigated with MCODE [36]. Pathway analysis was done with Enrichr [93]. Conservation of core interaction networks across tissues was investigated with GIANT 2.0 [94] (0.5 relationship confidence).

### Molecular docking and structural analysis

Molecular docking was performed using Hex [95], selecting "Shape+correlation+DARS" as correlation type and OPLS minimizations post processing. A total of 50.000 solutions were generated for each run. The PDB structures of pVHL [10] (PDB code 1LM8, chain V), human androgen receptor [71] (AR, PDB code 2AM9, chain A) and ELAVL [96] (PDB code 3H19, chain A) were retrieved from the RCSB database [97]. Docking models were ranked using CONSRANK [98] and the ten best scoring models visually inspected to select models fitting literature data best, i.e. concordance between interacting interfaces and described function.

### Petri net simulations

The effect of the most promising pVHL interaction impaired by the most recurrent pVHL mutations were inspected using the pVHL pathway Petri net model [32]. Loss of association with AR (Human androgen receptor, UniProt ID P10275) was modeled increasing the number of tokens describing MAPK activation, i.e. p_54. Similarly, the token number of places p_38, p39 and p_60 was increased to simulate loss of pVHL-dependent AKT-1 regulation. Mutations affecting pVHL interaction with ELAVL1, GSK3β were modeled through knock-outs of the corresponding transition, i.e. t_181, t_109. For each experiment, accumulation of tokens in specific places was inspected after 2,000 simulation steps and compared with the wild-type pVHL network.

## Supporting information

S1 Table Pathogenicity and stability predictions of pVHL mutations. PMUT: false/true represent the estimated risk for each mutation to be pathogenic. Align-GVGD: The prediction classes (C0, C15, C25, C35, C45, C55, C65) are for an estimated spectrum of pathogenicity with C65 most likely to interfere with function and C0 least likely.
(XLSX)

S2 Table Manually curated pVHL mutations. Mutations were ranked by number of affected patients.
(XLSX)

S3 Table Ring analysis of the most frequent pVHL mutations. Residue-residue interactions in wild type pVHL and the corresponding mutated residue. HBOND is for hydrogen bond, vdW is for van der Waals interaction, while PIPISTACK identifies cation π-π interaction. MC and SC refer to amino acid main and side chain respectively.
(XLSX)

S4 Table Short description of VHL-associated cancers. (XLSX)

S5 Table High quality pVHL interactors. List of manually curated pVHL interactors extracted from VHLdb.
(XLSX)

S6 Table Dataset of VHL mutations described as causative of a single phenotype. Mutations are mapped for affected interface and divided for each associated cancer.
(XLSX)

S7 Table List of mutations affecting pVHL interactors. Interactors binding interfaces are represented as yellow blocks according with bibliographic data.
(XLSX)

S8 Table PTM sites of pVHL. List of mutation affecting PTM sites and their predicted phenotype effects.
(XLSX)

S9 Table Supplementary literature. List of scientific works used to retrieve detailed information about VHL phenotypes.
(XLSX)

S1 File Result from MCODE analysis. Clusters of interactors of interactors associated to each pVHL surface.
(PDF)

S1 Fig Gene ontology enrichment of pVHL interactors. (TIFF)

S2 Fig Gene network analysis. Prediction of tissue-specific interactions among pVHL binding proteins.
(TIFF)

S3 Fig Overview of pVHL PTM sites. (TIFF)

S4 Fig Electrostatic surface of predicted pVHL molecular complexes. Electrostatic surfaces of pVHL in complex with ELAVL1 (up) and AR (down), with red representing negatively charged areas, while blue is for positive region. Proteins are alternatively presented as transparent views and rotated around vertical axis to better highlight complementary surfaces.
(TIFF)

S5 Fig Schematic representation of the analysis workflow. (TIFF)
