# Supplementary material for: Genotype-phenotype relations of the von Hippel-Lindau tumor suppressor inferred from a large-scale analysis of disease mutations and interactors
Source: PLoS Comput Biol. 2019 Apr 3;15(4):e1006478. doi: 10.1371/journal.pcbi.1006478 (PMC6464237; doi:10.1371/journal.pcbi.1006478)
Supplement: S1 File — Clusters of interactors of interactors associated to each pVHL surface. (PDF) [file pcbi.1006478.s010.pdf]

SURFACE A

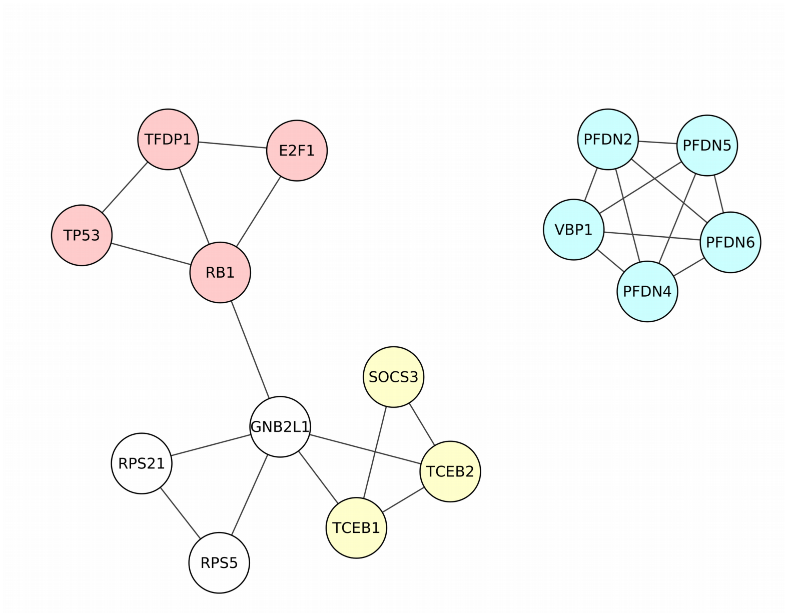

- cluster 1 (light blue): score 5; nodes 5; edges 10
- cluster 2 (pink): score 3.333; nodes 4; edges 5
- cluster 3 (yellow): score 3; nodes 3; edges 3

MCODE App Results

Parameters:
  Network Scoring:
    Include Loops: false Degree Cutoff: 2
  Cluster Finding:
    Node Score Cutoff: 0.2 Haircut: true Fluff: false K-Core: 2 Max. Depth from Seed: 100

Cluster	Score (Density*#Nodes)	Nodes	Edges	Node IDs
1	5	5	10	PFDN2, PFDN6, VBP1, PFDN4, PFDN5
2	3,333	4	5	RB1, TP53, E2F1, TFDP1
3	3	3	3	TCEB2, SOCS3, TCEB1

name	MCODE_Cluster	MCODE_Node_Status	MCODE_Score
PFDN4	Cluster 1	Clustered	4.0
VBP1	Cluster 1	Clustered	4.0
PFDN2	Cluster 1	Clustered	4.0
PFDN5	Cluster 1	Clustered	4.0
PFDN6	Cluster 1	Seed	4.0
TFDP1	Cluster 2	Clustered	1.6666666666666667
E2F1	Cluster 2	Clustered	2.0
RB1	Cluster 2	Clustered	1.6666666666666667
TP53	Cluster 2	Seed	2.0
TCEB1	Cluster 3	Clustered	1.6666666666666667
TCEB2	Cluster 3	Clustered	1.6666666666666667
SOCS3	Cluster 3	Seed	2.0
RPS21		Unclustered	2.0
RPS5		Unclustered	2.0
GNB2L1		Unclustered	1.2

SURFACE C

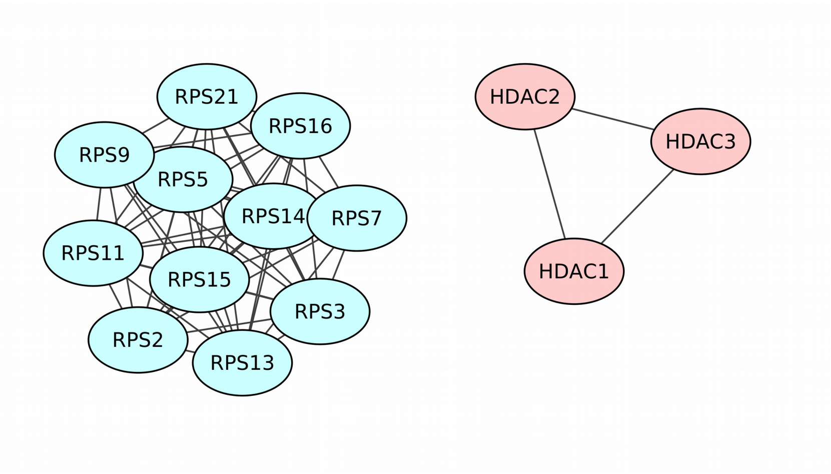

- cluster 1 (light blue): score 11; nodes 11; edges 55
- cluster 2 (pink): score 3; nodes 3; edges 3

MCODE App Results

Parameters:
Network Scoring:
Include Loops: false Degree Cutoff: 2
Cluster Finding:
Node Score Cutoff: 0.2 Haircut: true Fluff: false K-Core: 2 Max. Depth from Seed: 100

Cluster	Score (Density*#Nodes)	Nodes	Edges	Node IDs
1	11	11	55	RPS7, RPS13, RPS9, RPS15, RPS21, RPS5, RPS11, RPS14, RPS16, RPS3, RPS2
2	3	3	3	HDAC1, HDAC2, HDAC3

name	MCODE_Cluster	MCODE_Node_Status	MCODE_Score
RPS14	Cluster 1	Clustered	10.0
RPS5	Cluster 1	Clustered	10.0
RPS15	Cluster 1	Clustered	10.0
RPS9	Cluster 1	Clustered	10.0
RPS13	Cluster 1	Clustered	10.0
RPS7	Cluster 1	Clustered	10.0
RPS2	Cluster 1	Clustered	10.0
RPS3	Cluster 1	Clustered	10.0
RPS16	Cluster 1	Clustered	10.0
RPS11	Cluster 1	Clustered	10.0
RPS21	Cluster 1	Seed	10.0
HDAC2	Cluster 2	Clustered	2.0
HDAC1	Cluster 2	Clustered	2.0
HDAC3	Cluster 2	Seed	2.0

SURFACE B

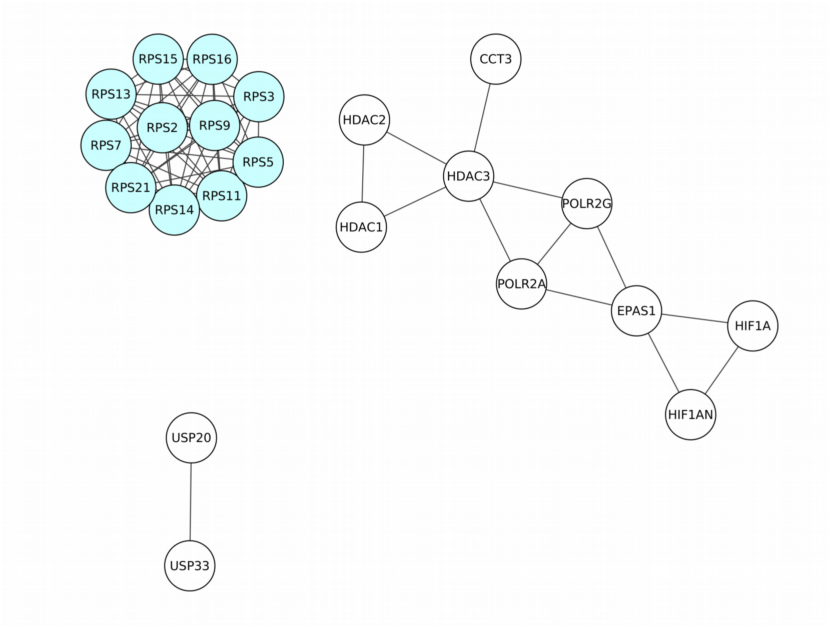

- cluster 1 (light blue): score 11; nodes 11; edges 55

MCODE App Results

Parameters:
  Network Scoring:
    Include Loops: false Degree Cutoff: 2
  Cluster Finding:
    Node Score Cutoff: 0.2 Haircut: true Fluff: false K-Core: 2 Max. Depth from Seed: 100

Cluster	Score (Density*#Nodes)	Nodes	Edges	Node IDs
1	11	11	55	RPS7, RPS3, RPS21, RPS5, RPS9, RPS2, RPS15, RPS11, RPS13, RPS16, RPS14

name	MCODE_Cluster	MCODE_Node_Status	MCODE_Score
RPS15	Cluster 1	Clustered	10.0
RPS2	Cluster 1	Clustered	10.0
RPS5	Cluster 1	Clustered	10.0
RPS3	Cluster 1	Clustered	10.0
RPS7	Cluster 1	Clustered	10.0
RPS14	Cluster 1	Clustered	10.0
RPS16	Cluster 1	Clustered	10.0
RPS13	Cluster 1	Clustered	10.0
RPS11	Cluster 1	Clustered	10.0
RPS9	Cluster 1	Clustered	10.0
RPS21	Cluster 1	Seed	10.0
POLR2A		Unclustered	1.6666666666666667
POLR2G		Unclustered	1.6666666666666667
HIF1A		Unclustered	2.0
HIF1AN		Unclustered	2.0
HDAC2		Unclustered	2.0
HDAC1		Unclustered	2.0
HDAC3		Unclustered	1.2
CCT3		Unclustered	0.0
USP33		Unclustered	0.0
USP20		Unclustered	0.0
EPAS1		Unclustered	1.2

C – TERMINAL REGION

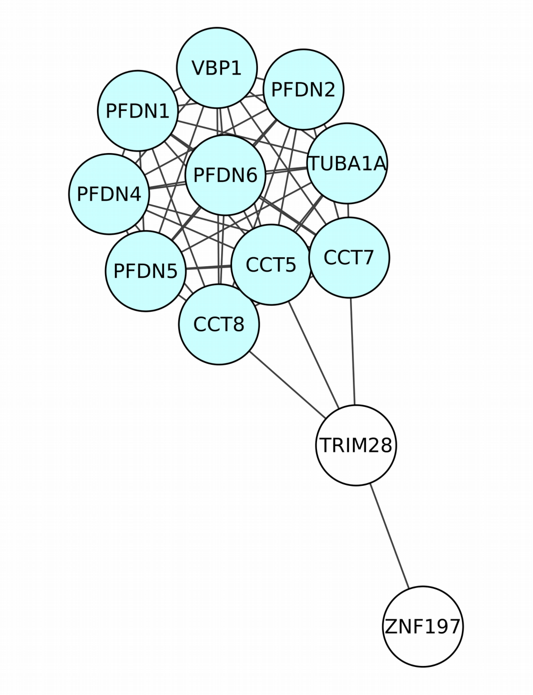

- cluster 1 (light blue): score 10; nodes 10; edges 45

MCODE App Results

Parameters:
Network Scoring:
Include Loops: false Degree Cutoff: 2
Cluster Finding:
Node Score Cutoff: 0.2 Haircut: true Fluff: false K-Core: 2 Max. Depth from Seed: 100

Cluster	Score (Density*#Nodes)	Nodes	Edges	Node IDs
1	10	10	45	PFDN5, PFDN1, CCT7, CCT8, PFDN2, PFDN4, PFDN6, TUBA1A, CCT5, VBP1

name	MCODE_Cluster	MCODE_Node_Status	MCODE_Score
PFDN4	Cluster 1	Clustered	9.0
PFDN2	Cluster 1	Clustered	9.0
CCT8	Cluster 1	Clustered	9.0
CCT7	Cluster 1	Clustered	9.0
PFDN5	Cluster 1	Clustered	9.0
VBP1	Cluster 1	Clustered	9.0
CCT5	Cluster 1	Clustered	9.0
PFDN6	Cluster 1	Clustered	9.0
PFDN1	Cluster 1	Clustered	9.0
TUBA1A	Cluster 1	Seed	9.0
ZNF197		Unclustered	0.0
TRIM28		Unclustered	3.0
