# Supplementary figures and images for: Genotype-phenotype relations of the von Hippel-Lindau tumor suppressor inferred from a large-scale analysis of disease mutations and interactors
Source: PLoS Comput Biol. 2019 Apr 3;15(4):e1006478. doi: 10.1371/journal.pcbi.1006478 (PMC6464237; doi:10.1371/journal.pcbi.1006478)

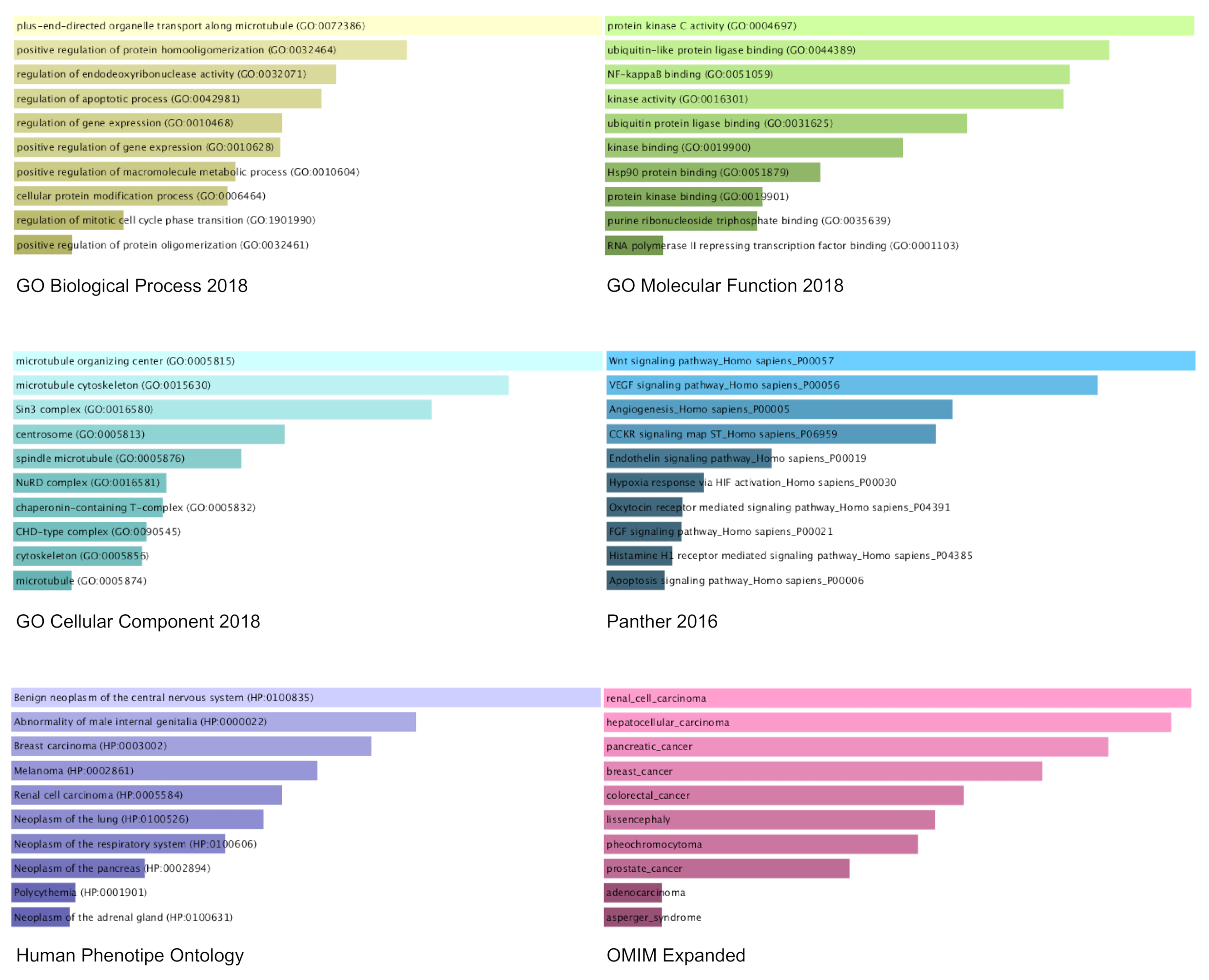

Supplement: S1 Fig — (TIFF) [file pcbi.1006478.s011.tiff]

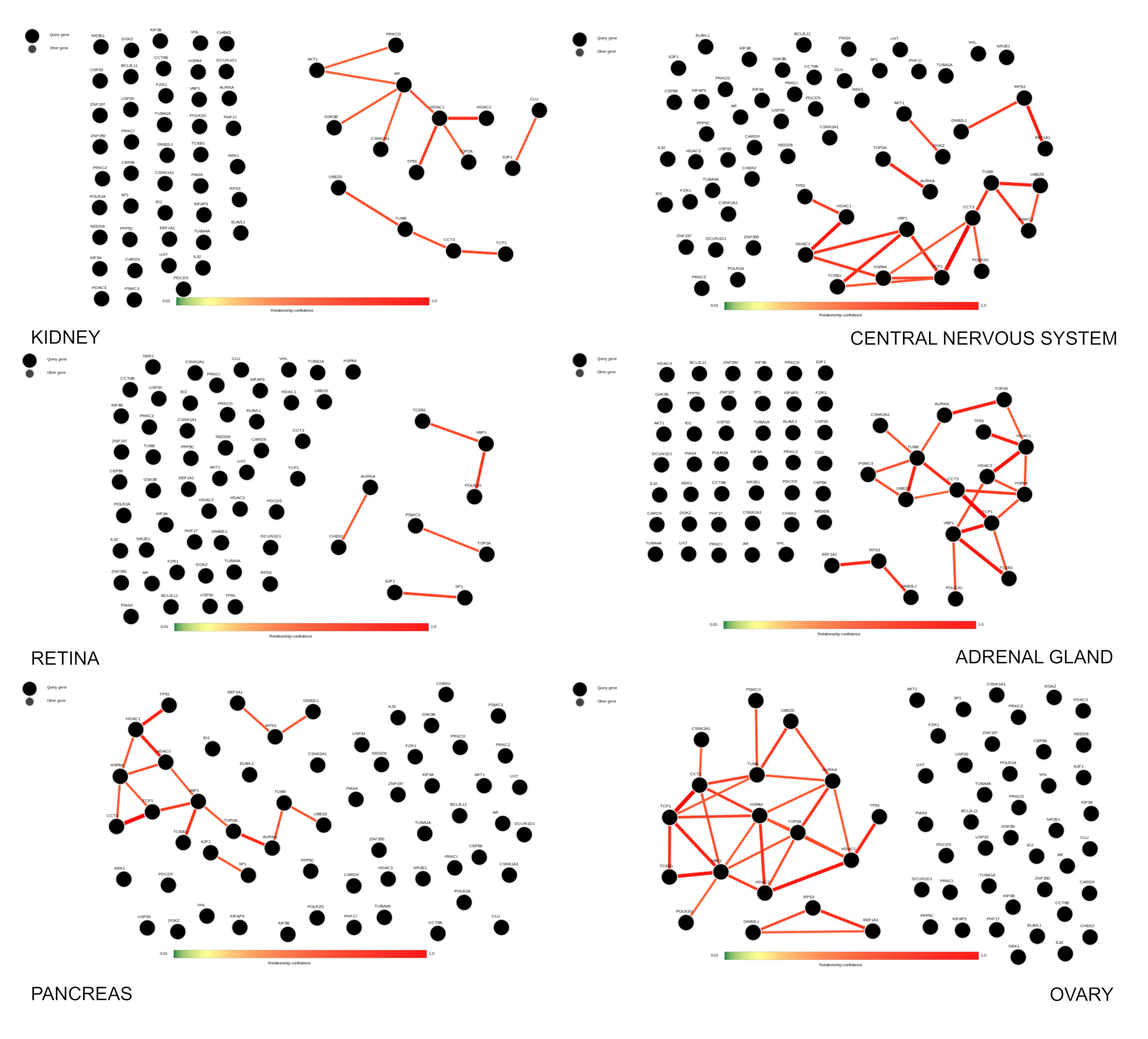

Supplement: S2 Fig — Prediction of tissue-specific interactions among pVHL binding proteins. (TIFF) [file pcbi.1006478.s012.tiff]

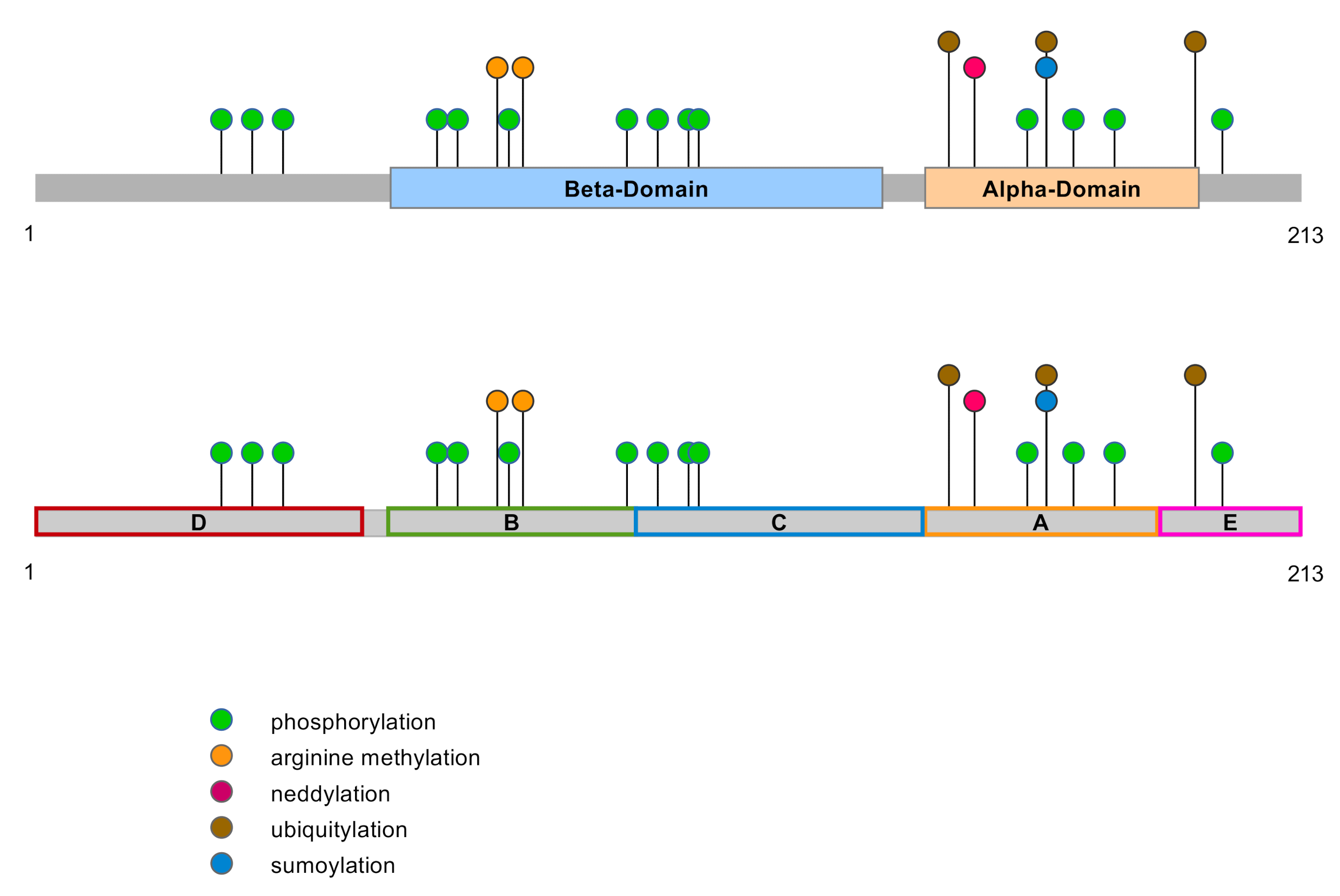

Supplement: S3 Fig — (TIFF) [file pcbi.1006478.s013.tiff]

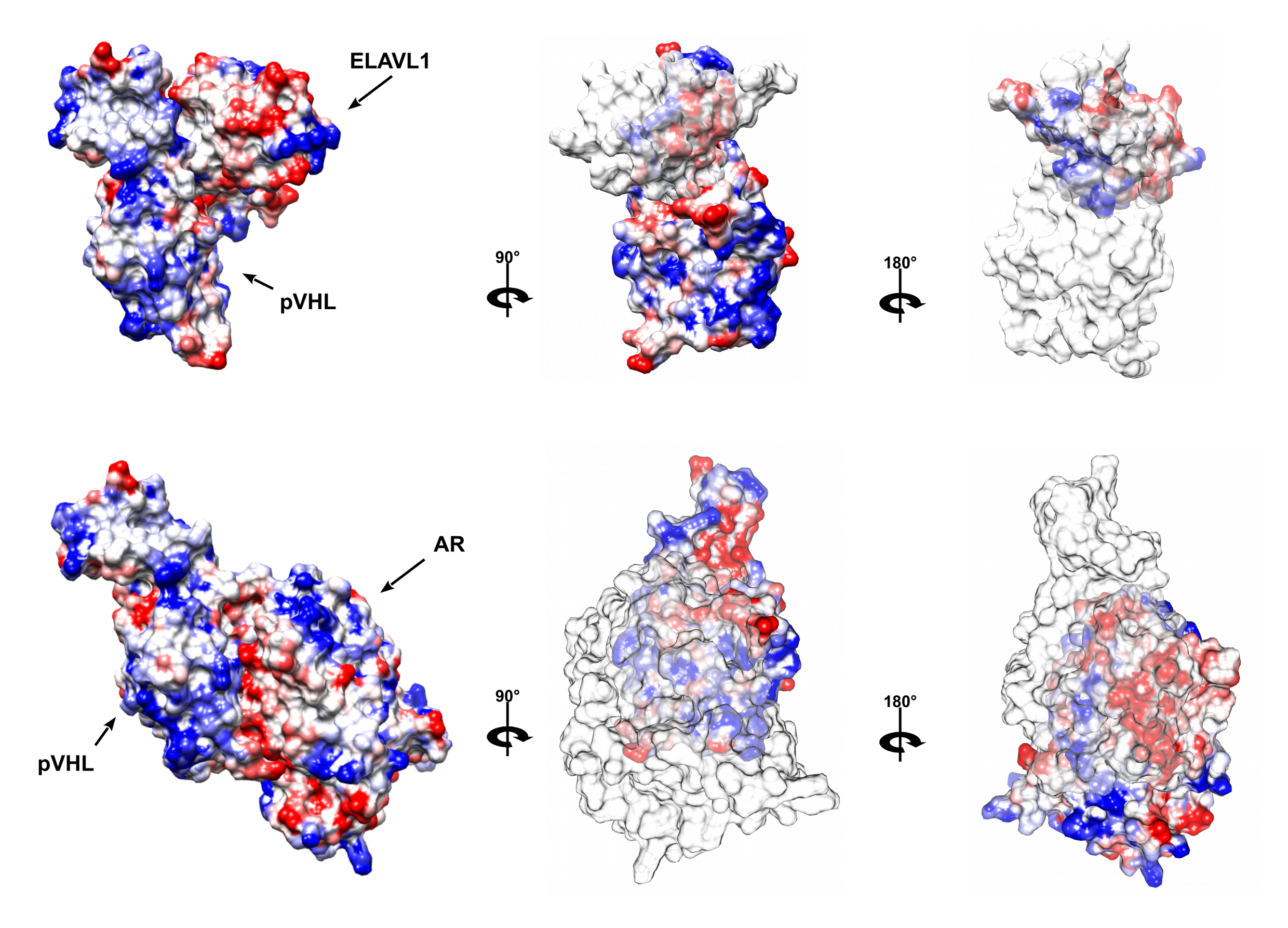

Supplement: S4 Fig — Electrostatic surfaces of pVHL in complex with ELAVL1 (up) and AR (down), with red representing negatively charged areas, while blue is for positive region. Proteins are alternatively presented as transparent views and rotated around vertical axis to better highlight complementary surfaces. (TIFF) [file pcbi.1006478.s014.tiff]

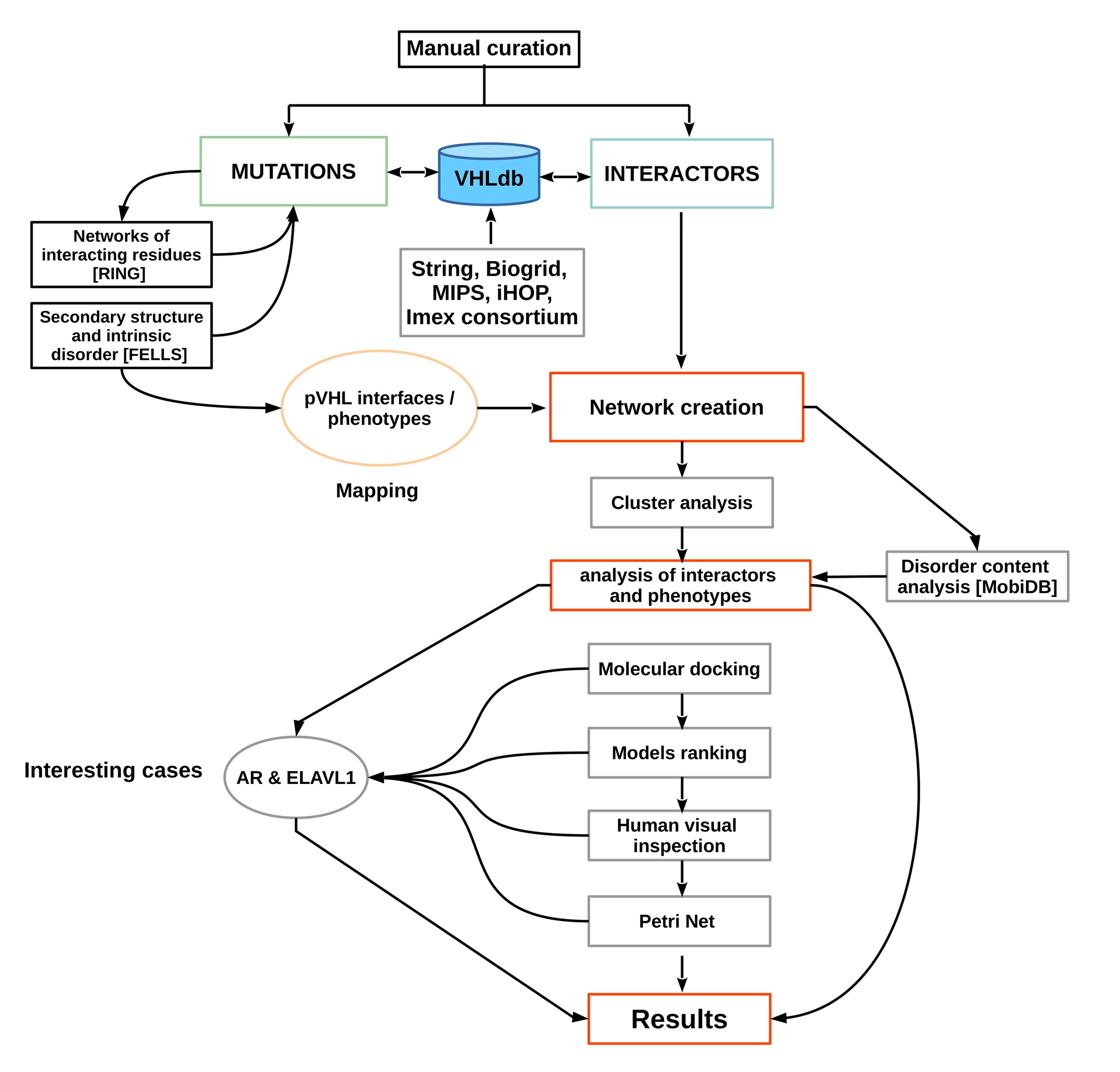

Supplement: S5 Fig — (TIFF) [file pcbi.1006478.s015.tiff]
